# Airflow modelling predicts seabird breeding habitat across islands

**DOI:** 10.1111/ecog.05733

**Published:** 2021-11-21

**Authors:** Emmanouil Lempidakis, Andrew N. Ross, Luca Börger, Emily L. C. Shepard

**Affiliations:** Dept of Biosciences, Swansea Univ., Swansea, UK; School of Earth and Environment, Univ. of Leeds, Leeds, UK; Dept of Biosciences, Swansea Univ., Swansea, UK; Centre for Biomathematics, College of Science, Swansea Univ., Swansea, UK; Dept of Biosciences, Swansea Univ., Swansea, UK

**Keywords:** climate change, computational fluid dynamics, distribution, flight, habitat use, seabird, wind

## Abstract

Wind is fundamentally related to shelter and flight performance: two factors that are critical for birds at their nest sites. Despite this, airflows have never been fully integrated into models of breeding habitat selection, even for well-studied seabirds. Here, we use computational fluid dynamics to provide the first assessment of whether flow characteristics (including wind speed and turbulence) predict the distribution of seabird colonies, taking common guillemots *Uria aalge* breeding on Skomer Island as our study system. This demonstrates that occupancy is driven by the need to shelter from both wind and rain/wave action, rather than airflow characteristics alone. Models of airflows and cliff orientation both performed well in predicting high-quality habitat in our study site, identifying 80% of colonies and 93% of avoided sites, as well as 73% of the largest colonies on a neighbouring island. This suggests generality in the mechanisms driving breeding distributions and provides an approach for identifying habitat for seabird reintroductions considering current and projected wind speeds and directions.

## Introduction

Reproductive success is closely linked to the physical characteristics of breeding sites in many taxa (Birkhead et al. 1985, Harris et al. 1997). In colonial animals, breeding sites can represent the nexus of reproductive activity for tens of thousands of individuals (Buckley and Buckley 1980). There is therefore a clear need to establish what drives colony location in order to identify the availability of breeding habitat and predict how areas differ in quality, now and in the future (Frid and Dill 2002, Parmesan and Yohe 2003, Jetz et al. 2007, Sorte and Thompson III 2007, Dunlop 2009, Chambers et al. 2011, Rushing et al. 2020).

Over 95% of seabirds are colonial breeders (Rolland et al. 1998). Seabirds are also more at risk than other comparable groups of birds, with a widespread decline in populations due to commercial fisheries, pollution, habitat change and the introduction of invasive predators (Croxall et al. 2012). In some cases, this has led to entire breeding colonies being lost (Jones et al. 2008, Brooke et al. 2018). Thus, conservation practitioners need to know where to focus restoration efforts e.g. by decoy deployment and acoustic attraction to re-seed breeding activity (Jones and Kress 2012). This is crucial given that there will always be a fitness cost associated with breeding in suboptimal habitat (Brown 1969). However, while a wide range of studies have analysed breeding site characteristics in seabirds (Buckley and Buckley 1980, Birkhead et al. 1985, Harris et al. 1997, Keslinka et al. 2019) and compared them with available habitat (Clark et al. 1983), we are unaware of any that have successfully applied predictions from one site to another (cf. Aarts et al. 2008). Achieving such generality and transferability is paramount for conservation success, as well as being of fundamental ecological interest.

The tendency of seabirds to breed on offshore islands and/or coastal cliffs has been attributed to the need to reduce exposure to terrestrial predators and be close to feeding areas (Cody 1969, Buckley and Buckley 1980). Nonetheless, for cliff-nesting species, it is clear that not all cliffs are equal, as colonies tend to be clumped, with great swathes of cliff habitat left empty (Harris et al. 1997). Indeed, cliffs should vary in their accessibility to terrestrial predators (primarily through variation in slope angle), as well as the availability of suitable breeding ledges, with species varying in their need for different ledge characteristics according to their body size and nest-building habit (Squibb and Hunt 1983, Birkhead et al. 1985).

There are also compelling reasons why airflows should affect breeding habitat preferences, particularly for groups such as seabirds, which are exposed to strong flows. Wind can affect the risk of eggs/birds being displaced from the nest (Hamer et al. 2001, Newell et al. 2015), as well as influencing exposure to rain (particularly in cliff-nesting species) and heat stress (through evaporative heat loss, Oswald and Arnold 2012), both of which can cause mortality (Hamer et al. 2001). Wind also has a strong influence on flight capacity. In common guillemots *Uria aalge* and razorbills *Alca torda*, 60% of attempts to land at their cliff nests were found to fail in a strong breeze, which may have consequences for the ability to provision chicks as well as adult energy budgets (Shepard et al. 2019). This raises the possibility that site selection might be influenced by the ability to land. Indeed, frigate birds, *Fregata magnificens*, nest in relatively wind-still areas, despite the potential benefits of wind in aiding heat loss for this tropical species. This most likely reflects the difficulties that frigate birds experience in flying close to the nest in high winds (Diamond 2002). While a reduction in wind speed would be beneficial for their flight capacity, other factors may also influence landing ability, including turbulence, as this requires compensatory manoeuvring (cf. Ravi et al. 2015, Cheney et al. 2020). Up- or downdrafts may also be relevant, for instance, downdrafts could even facilitate landing in fast-flying birds such as guillemots, by reducing their ground speed as they ascend to their cliff-nesting sites.

Despite the potential importance of wind, there is a notable lack of information on the precise wind flow characteristics associated with colony presence and absence (though see Burger and Lesser 1978), which reflects the difficulties of making measurements over complex, often steep terrain. Here, we use an open-source model based on computational fluid dynamics (CFD) to estimate a range of airflow characteristics that relate to shelter (including the magnitude of the wind and the horizontal component) and factors that could specifically impact landing ability (the vertical wind component, wind gusts (short peaks in wind speed) and turbulence (rapid unsteadiness in wind speed and direction) (Ravi et al. 2015, Shepard et al. 2019). We then assess whether wind components can predict the presence and absence of common guillemots *Uria aalge* breeding on Skomer Island, UK. Our specific objectives were to: 1) assess whether airflows associated with the prevailing wind direction predict site selection (patterns of presence and absence) and habitat quality (colony density) and then 2) test our model of habitat selection by predicting colony presence and absence on a neighbouring island. This test is considerably stronger than standard cross-validation but rarely performed (Aarts et al. 2008). Finally, 3) we run airflow simulations to quantify the wind conditions that breeding guillemots are exposed to with changes in wind direction. Overall, our approach should provide insight into the conditions birds select and avoid in the prevailing wind, and why, and the ‘penalty’ they suffer in terms of the adverse conditions they are exposed to if the wind direction changes, either over the short term or as part of larger-scale climatic shifts (Young et al. 2011, Young and Ribal 2019).

## Material and methods

The first step in our study was to predict the distribution of guillemot colonies on Skomer Island (51°44'27.1"N, 5°17’66.8"W) and apply this model to predict colony occurrence on the neighbouring island of Skokholm. The distribution of guillemots on Skomer was taken from the 2015 guillemot breeding bird survey (Stubbings et al. 2015, Supporting information). This provides counts of breeding birds within 71 adjoining horizontal sections, with individual survey sections being delineated by topographical features. In order to assign slope angles and wind parameters to each section, the survey was digitized in ArcMap 10.5.1 (ESRI, Redlands, California), and horizontal section boundaries were mapped onto a digital elevation model (DEM) (50-cm resolution retrieved from Lle Geo-Portal <http://lle.gov.wales>).

Each section was then defined vertically with a minimum height of 10 m a.s.l. to account for variation in tide height (maximum tide height ~5 m on the day the DEM was produced), maximum wave height (taken to be 3 m) and the minimum distance above water that birds tend to nest, taken as 2 m (Harris et al. 1997). The maximum height of each section was taken as 15 m from the top of the cliff, which was the mean upper limit of nests for three major colonies (Cole et al. 2019).

A similar approach was taken to digitize the distribution of guillemots breeding on Skokholm Island using the 2018 breeding bird survey (Brown and Eagle 2018) (Supporting information). However, because the elevation of Skokholm’s cliffs is much lower, the minimum distance from the top of the cliffs was set at 7 m (this was arrived at in consultation with the island wardens). The small proportion of occupied cliffs that did not satisfy this threshold was not mapped (~9% of the coastline at 7 m height). In cases where estimated bird numbers were given in relation to a single point on the map, we used a minimum section length of 30 m of coastline, unless ascribing this width to adjacent colonies would have resulted in unoccupied sections of <30 m, in which case we assigned a section of 30–50 m in length. This approach resulted in 91 sections, with 35 being colonised (Supporting information).

### Modelling of wind conditions

Wind conditions were modelled using the computational fluid dynamics (CFD) package OpenFOAM (<www.open-foam.org> ver. 5.x). OpenFOAM is widely used for modelling atmospheric boundary layer flows (e.g. in the wind energy industry) and has been extensively validated over a similarly steep island (Bechmann et al. 2011).

The initial coarse model domain was 5300 × 5000 × 1000 m, with a horizontal resolution of 20 m and a vertical resolution of 10 m. The bottom boundary represented the surface of the island which was taken from a DEM of Skomer with a 2-m resolution (Lle Geo-Portal <http://lle.gov.wales>). After establishing the initial mesh, the tool snappyHexMesh in OpenFOAM was used to incorporate the DEM, refining initial mesh cells close to the surface up to three times and performing standard mesh quality tests and corrections (<https://cfd.direct/openfoam/user-guide/v6-snappyhexmesh/>). This resulted in a finer resolution close to surface of 2.5 m in the horizontal and 1.25 m in the vertical. Simulations were completed when convergence was achieved using a steady-state incompressible solver with a k-ε turbulence closure scheme (using standard settings).

Simulations of the wind over Skomer and Skokholm were run for the prevailing SW direction (see the Supporting information for summary of wind data) assuming that birds would be most likely to select breeding habitat in relation to these conditions. Wind simulations were also run for north-westerly (NW), north-easterly (NE) and south-easterly (SE) winds around Skomer in order to assess the penalty that birds face when the wind direction changes. The initial wind speed was set to 10 m s^−1^ at 20 m height. The following airflow characteristics were extracted from the model output at 2 m normal to the ground surface (this height was selected to estimate the airflow conditions that birds would be exposed to close to their breeding cliffs): the two horizontal and vertical wind components (U_0, U_1 and U_2 respectively), mean wind speed (MeanU), turbulent kinetic energy (TKE, a measure of the absolute wind unsteadiness), dynamic pressure (P, given relative to the background hydrostatic pressure set in the simulations), kinematic viscosity (Nut) of the air medium and turbulence dissipation rate (ε). These outputs were further used to estimate horizontal wind speed, wind gusts and turbulence intensity (TI, the ratio of turbulence to wind speed, indicating the importance of wind variability with respect to the mean flow strength).

Each of the four OpenFOAM simulations (i.e. for NW, NE, SE and SW wind directions) resulted in 76 908 data points across all cliff sections. Each airflow parameter was then reduced to the following summary statistics within each 3-D section area: median, interquartile range (IQR) and skewness. Median statistics were used to identify the strength of each wind parameter within a section. The IQR values identify the variability or gradient of each wind parameter within a section. High skewness statistics for horizontal wind speed (skewed right) correspond to shelter.

### Statistical analysis

The complete set of 27 airflow parameters was tested for collinearity by producing a correlation hierarchy table for each initial wind condition. Highly correlated terms (Pearson correlation coefficient ≥ 0.7) were removed from the analysis, making sure that statistics from at least one airflow parameter representing exposure, wind strength or turbulence were maintained, leading to the inclusion of 15 wind parameters. The mean slope angle per section was added to the total set, together with the logarithmic area of each section (as an offset), and parameters were standardized using the MuMin package (Barton and Barton 2015) ver. 1.43.17.

We considered that it would be stretching the data to model the density or number of breeding birds as a continuous variable, particularly as the extent of each section did not correspond with the beginning or end of occupied/unoccupied areas. We therefore ran separate models, with colony presence defined as 1) the presence of any breeding birds, 2) the 10 largest (n ≥ 592 individuals) or 3) the 11 densest colonies (density ≥ 0.835 m^−1^) (thresholds were selected by visually identifying clear breakpoints; however, we also conducted a sensitivity analysis to evaluate the robustness of our results to the choice of ‘largest colonies’). For the latter two categories, areas with breeding birds that did not fall into either the largest or densest categories were excluded from the modelling. This allowed us to test what distinguishes the highest quality habitat from the lowest quality habitat (assuming these correspond to the largest/densest areas and areas with a complete lack of breeding birds, respectively). The excluded areas represented 23.5% and 27.7% of all breeding birds for the largest and densest classifications and resulted in trained datasets of 43 and 44 sections for the largest and densest datasets.

A two-step approach was used to build the global model and identify the final, best-fitting models. First, to reduce the large number of covariates (as compared to the number of data points), a random forest classifier was fitted, using the package randomForest (Liaw and Wiener 2002) ver. 4.6.14. The 10 most important terms were then included to build the global logistic regression model. This parameter set was further simplified using the dredge function (MuMin package, Barton and Barton 2015), to perform stepwise Bayesian information criterion (BIC) selection, penalising for model size to highlight the terms with the strongest effects. In the case of the models for largest and densest colonies in an NW wind, the number of terms in dredge had to be gradually reduced to eight and seven, respectively, to prevent fitting models with probabilities of zero and one. The simplest model among the top models with a difference in BIC ≤ 2 was selected as the best final model. This was compared to a model of mean slope and orientation, where sections were defined as either windward or leeward with respect to the prevailing SW wind. The same method of model selection was used to identify the top simplest model across the combinations of these two parameters. In order to interpret the output of the orientation model, we also estimated the total mean solar radiation (W h m^2^) during the breeding season in 2015 using ArcMap 10.7 (assuming generally clear sky).

The final models were assessed for spatial autocorrelation using the DHARMa package (Hartig 2020) ver. 0.3.3 and for goodness of fit using the McFadden (McFadden 1973) pseudo *R* ^2^. Values between 0.2 and 0.4 were considered as very satisfactory (Naugle et al. 1999). Model performance was also evaluated in terms of overall accuracy (OA), true skill statistic (TSS), sensitivity and specificity (Allouche et al. 2006, Börger and Nudds 2014). The effect size of each predictor included in the final model was determined by computing the odds ratio.

The previous steps were repeated for all four initial wind directions, with three logistic regression models of colony presence/absence implemented per direction, in order to identify links between wind and slope that were robust to different colony definitions.

Finally, to predict the distribution of colonies on Skokholm, we applied the model of the 10 largest colonies on Skomer, using conditions experienced in the prevailing SW wind, and converting the predicted odds into presence/absence by selecting the cut-off value which maximises TSS (Liu et al. 2011, Börger and Nudds 2014).

All statistical analyses were conducted in R (<www.r-project.org>) ver. 3.6.3 and RStudio (RStudio Team 2015) ver. 1.1.463.

## Results

### Predicting colony distribution on Skomer Island

**Figure 1:**
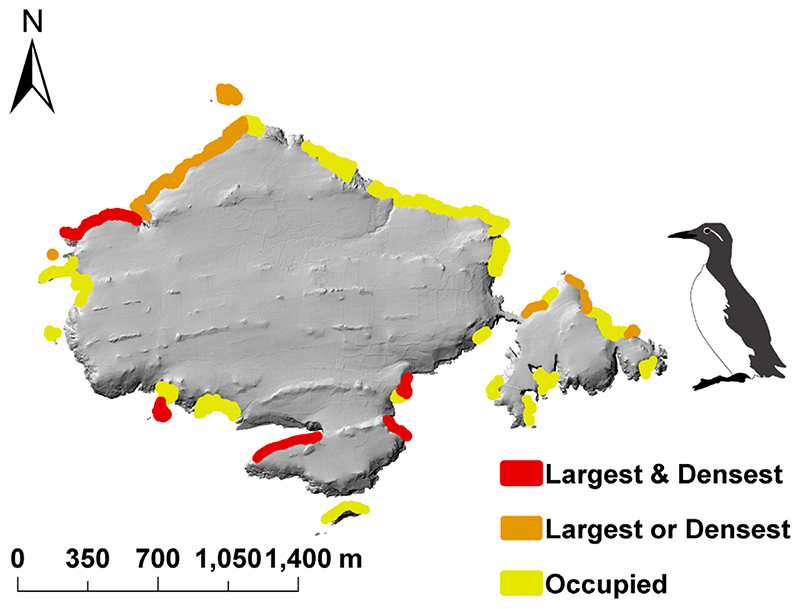
The guillemot survey sections on the cliffs of Skomer and the distribution of breeding guillemots. Areas classified as both densest and largest are indicated in red (n = 7 sections). Areas that were identified as among either the largest (n = 3) or densest (n = 4) are indicated in orange. Residual occupied areas are indicated in yellow.

Wilcoxon rank-sum tests for all colony classifications revealed statistically different slope angles between colonies and non-colonies on Skomer Island (taking presence as any positive count, W = 611 724 994, p < 2.2 ×10^-16^; as the largest colonies, W = 199 340 779, p < 2.2 ×10^-16^; or the densest, W = 71 820 756, p < 2.2 ×10^-16^). Unoccupied sections were generally less steep than occupied sections (median slope angles 45° and 51°, respectively), and the densest and largest colonies were associated with the steepest cliffs (median = 68.5°, Supporting information). Slope angle varied with cliff orientation, with mean slope angle being lowest for cliffs with S and SE orientations. Cliffs facing SW have relatively high mean slope angles (Supporting information). Despite this, most occupied sections were orientated away from the prevailing SW wind (Fig. 1).

Colony presence and absence on Skomer was predicted well by wind parameters and slope angle, with the simplest top model correctly identifying 80% of the colonies and 93% of avoided sites for the prevailing SW wind, where presence was taken as the 10 largest colonies (see Table 1 for the full list of model outputs). While mean slope angle was included in two of three models for SW, NW and NE wind directions, airflow parameters always had a higher effect size (Supporting information). A sensitivity analysis confirmed that the parameters in the final models were largely consistent, irrespective of whether colony presence was taken as the 11, 12, 13, 14 or 15 largest colonies (Supporting information).

**Figure 2:**
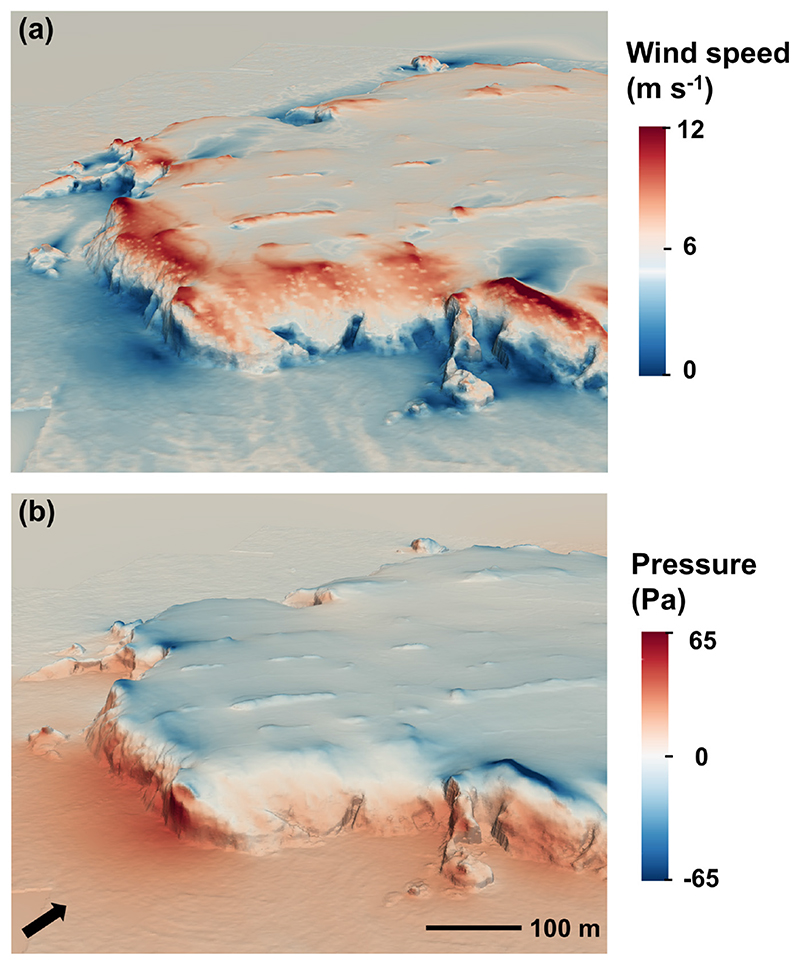
The OpenFoam model output of (a) wind speed and (b) pressure, over a windward cliff (‘Skomer head’) with SW wind (denoted with a black arrow). In the lower parts of the cliffs, the wind is blocked, resulting in high-pressure regions where flow is decelerated. Closer to the top (55–60 m a.s.l.), the flow is accelerated, generating areas of low pressure. This results in highly variable pressure values on windward cliffs. On leeward cliffs, flow separation occurs, viscous forces take over, and areas of consistently low pressure are generated that are not associated with high wind speeds.

A narrow set of airflow parameters was identified as significant in predicting colony presence across wind directions, and there was broad agreement between the airflow characteristics identified for any wind direction, irrespective of the way colonies were classified (Table 1). Pressure statistics were included in all SW models, with colonies having a lower median pressure and pressure gradient (Table 1). Both these factors are linked to lower exposure (Fig. 2, Supporting information), a relationship that was confirmed by a simple model predicting presence according to whether sections had a windward or leeward orientation in relation to the prevailing SW wind and slope angle. This model of orientation and slope angle performed similarly to the model with pressure and slope for the largest colonies (Supporting information). Given the importance of exposure in determining colony presence, it was surprising that no wind speed parameters were included in the simplest top models for SW winds.

**Figure 3:**
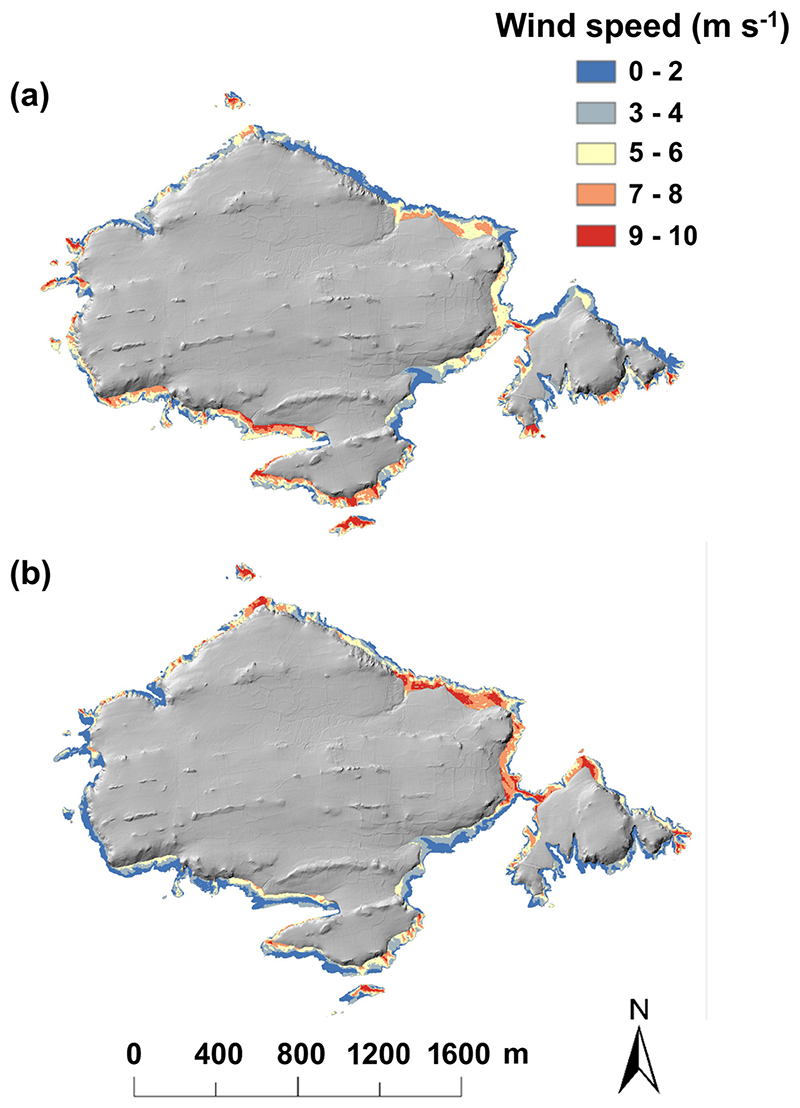
Modelled horizontal wind speeds on the cliffs of Skomer. Modelled wind speeds under (a) SW and (B) NE wind direction. Mean wind speeds were reduced on leeward cliffs, increased on windward cliffs and reached their highest estimates at the crests, as expected. Winds were modelled 2 m normal to the surface, and the mapped area was constrained by the 4-m and 40-m elevation contours.

Colonies were associated with higher turbulence compared to unoccupied sites, particularly in NW and NE winds (Table 1). In NE winds, colonies experienced both higher wind speeds (Fig. 3) (positive horizontal median for any bird presence and negative horizontal skewness for the largest/densest colonies) and higher turbulence (here TKE estimates in the largest/densest colonies). In fact, airflow models of colony location performed well across all modelled wind directions except for SE winds, indicating that colonies are characterised by particular sets of flow characteristics in most scenarios. For SE winds, only one colony classification yielded a reasonable model fit (McFadden^41^ *R* ^2^ ≥ 0.2, Table 1), and here colony presence was predicted by slope angle alone (Table 1).

Across the different colony classifications, the pseudo *R* ^2^ was lowest in models of any occupancy (SW 0.28, NW 0.24, SE 0.12, NE 0.38) compared to those predicting the 10 largest colonies (SW 0.59, NW 0.58, SE 0.32, NE 0.74) or the 11 densest colonies (SW 0.17, NW 0.40, SE 0.15, NE 0.67) (Table 1, Supporting information). The overall accuracy (OA) and true skill statistics (TSS) followed the same general trend, being highest for the 10 largest colonies (Table 1, Supporting information). The sensitivity tended to be somewhat lower than specificity for any presence (sensitivity: SW 0.63, NW 1.00, SE 0.21, NE 0.60 and specificity: SW 0.69, NW 0.21, SE 0.96, NE 0.84), but increased for the largest and densest colonies indicating a better ability to predict true presence compared to true absence in these cases.

### Predicting colony distribution on Skokholm

**Figure 4:**
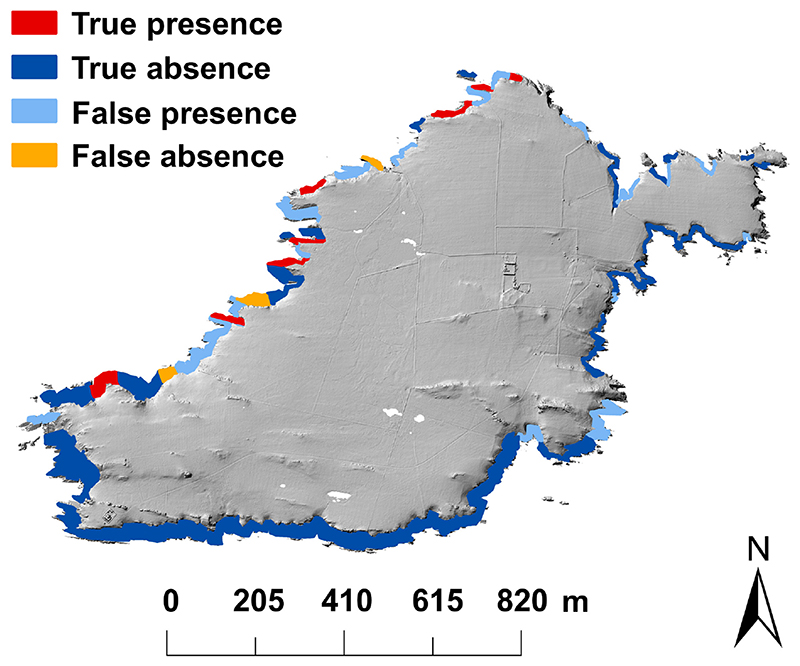
Predicted distribution of guillemot colonies on Skokholm. The distribution was predicted using the model of the largest colonies on Skomer and the wind field on Skokholm as predicted under the prevailing SW wind.

The model predicting the largest colonies on Skomer for SW winds also performed well when applied to the island of Skokholm (Fig. 4), correctly predicting the distribution of ~73% of the largest colonies (8 of 11) and ~63% of unoccupied cliff sections (35 of 56), which corresponds to ~80% of the total unoccupied area (60 863 m^2^ of 76 259 m^2^). Model performance, although lower than the models on Skomer, was satisfactory with an OA of 0.64 and TSS of 0.35. The model predicting the largest guillemot colonies on Skomer according to orientation and slope performed better than the airflow model when applied to Skokholm, correctly predicting all the largest colonies and ~71% (40 of the 56) unoccupied sections, with an OA of 0.76 and TSS of 0.71.

## Discussion

Wind regimes are changing in terms of the mean strength and the frequency of extreme weather events (Young et al. 2011, Young and Ribal 2019). Yet most research on how wind affects seabirds has focused on their at-sea behaviour (though see Weimerskirch et al. 2012, Newell et al. 2015, Schrimpf and Lynch 2021). We modelled the airflows around our study site to test the role of different wind characteristics in breeding habitat selection and showed that a simple model of airflows and slope angle performed very well for common guillemots, even when applied to the separate island of Skokholm. Both this approach and a model of cliff orientation showed that birds select sheltered, leeward sites. Indeed, a model of orientation and slope performed better in predicting the distribution of breeding birds on Skokholm. Nonetheless, the orientation model cannot tell us what birds are sheltering from. We demonstrate how airflow modelling can provide mechanistic insight into the factors driving habitat selection, as well as quantify the airflow conditions that birds are exposed to in different wind directions.

While colony presence on Skomer was predicted by low exposure to the prevailing SW wind, surprisingly, it was not predicted by low wind speed. Instead, areas of low pressure and low pressure variability were powerful predictors of cliff occupancy, both of which are characteristic of sheltered areas. Birds are less likely to be responding to the pressure values directly, than the exposure that they are correlated with, because the absolute difference in pressure values between occupied and unoccupied sites is equivalent to the changes experienced over the diurnal cycle.

The absence of wind speed from models of guillemot distributions can be explained by 'orographic blocking', a process whereby windward cliffs can block the oncoming flow, with the blocking effect increasing with cliff height and slope. This produces low wind speeds over large parts of windward cliffs, bar the top where flow is accelerated (Fig. 2, Supporting information). Low wind speeds therefore occur in both leeward and windward sites. The fact that birds avoid the latter, even though they tend to occur on suitably steep slopes, suggests that shelter from rain (Cleeland et al. 2020), and/or wave action during storms, is at least as important as low wind speed in determining site use (the former is much more frequent in the breeding season, Newell et al. 2015). Therefore, while the ability to fly, and specifically to land, at the nesting site may affect habitat selection, it is also influenced by the need to shelter young from rain as well as storm surges. Nevertheless, flight capacity may be most important for species such as large albatrosses, which require relatively high winds to take-off and therefore may be constrained to nest in exposed areas, despite the intuitive benefits of shelter for chicks across species in temperate or cold climates.

Heat stress is an important determinant of chick survival in some systems (Oswald et al. 2008), but it seems unlikely to be the primary driver of habitat selection here, as while occupied cliffs tend to experience lower solar radiation than unoccupied sites, three of the largest and four of the densest colonies on Skomer still experience high degrees of insolation (Supporting information).

The fact that our models performed better in correctly predicting the largest/densest colonies, compared to the presence of any breeding birds, suggests that they work best in predicting high-quality habitat. Previous studies have shown that breeding success increases with the density of breeding pairs (Birkhead 1977, Harris et al. 1997). Areas that can support larger numbers are therefore of higher quality. Such areas have previously been described in terms of the number of walls, slope and width of the ledge where the egg was incubated and distance from the top of the cliff (Birkhead et al. 1985, Harris et al. 1997). The ability to predict high-quality breeding habitat without such fine-scale topographical information is advantageous, as it allows habitat quality to be predicted in remote and inaccessible sites. Our models confirm the value of steep slopes to guillemots, which offer the possibility of breeding in high densities with better protection from predators (Birkhead et al. 2018), as well as easier access to the sea when chicks jump from their nests (Gilchrist and Gaston 1997, Berger 2008). Nonetheless, steep cliffs with a south-westerly orientation are avoided, even though they are widely available.

Our model may be more accurate than the frequency of false positives suggests, as cliffs that are currently unoccupied may still offer suitable habitat that only becomes used when existing sites reach full capacity. Indeed, photographs of the breeding cliffs on Skomer from the 1930s provide evidence that numbers were much higher historically (Harris 1991), and whole island counts undertaken since 1963 demonstrate that numbers have been increasing since then (Meade et al. 2013). Sites identified as ‘false positives’ therefore represent potentially high-quality habitat that could be suitable for re-establishing breeding colonies in systems where breeding activity occurs in a fraction of the former range. Similarly, our method could be used to model the specific wind strengths that areas are exposed to in storm conditions in systems where that can affect breeding success (Newell et al. 2015). Finally, in cases where populations are increasing, our approach could be extended to see whether airflow characteristics can predict colony growth rates or areas most likely to be expanded into.

While guillemots preferentially breed in areas that are not exposed to the prevailing wind, they cannot shelter from all wind directions. Winds diametrically opposed to the prevailing direction (here NE winds) will be problematic for any species seeking sheltered sites. The penalty of exposure to NE winds for the 10 largest colonies on Skomer was a ~10% increase in mean wind speed compared to the same at-sea wind speeds from the SW. How this might impact birds will vary with the magnitude of the wind when it comes from a different direction. Nonetheless, our results highlight that colonies experience increased exposure from changes in wind direction, independent of wind speed. Increases in wind speed, as already observed in the North Atlantic and other areas (Young et al. 2011, Young and Ribal 2019), should therefore be most detrimental at the nest when accompanied by a change in wind direction (Weimerskirch et al. 2012).

A further challenge potentially faced by birds on Skomer in NE and NW winds is increased turbulence. The absolute levels of turbulence that birds experience in SW winds are low because the wind speeds themselves are low. However, in NE winds of the same magnitude, birds experience both stronger winds and increased turbulence. Disturbed wind fields are known to have a critical impact on the safety of aircraft landings, but almost nothing is known about how turbulence affects landing in birds (cf. Chang et al. 2016). Nonetheless, it seems likely that this will only make landing more difficult, resulting in more aborted attempts and higher associated costs, particularly in species such as guillemots with low manoeuvrability (Shepard et al. 2019).

Overall, therefore, our results show that CFD can be used to predict occupancy directly or combined with other approaches, such as the use of orientation or wind fetch (Schrimpf and Lynch 2020), to provide insight into the mechanisms underpinning habitat selection. The approach we have developed in this study is therefore likely to be applicable to a range of seabirds, in terms of assessing drivers of habitat selection and the tradeoffs that some animals face. Indeed, CFD is particularly well-suited to modelling habitat selection in seabirds, as marine and coastal environments experience some of the most extreme wind conditions (Wiley and Wunderle 1993), and wind fields also tend to be more uniform ahead of islands. Although CFD models tend to be computationally expensive, they are widely used, with many open-access platforms available and can be run on personal computers (depending on the area size and resolution). Our approach can therefore easily be replicated with even basic knowledge of geographic information system and remote sensing, in combination with broadly available survey data. A key future challenge will be to test this approach over larger areas. Combining airflow modelling with data on breeding success in a range of conditions (cf. Cleeland et al. 2020) will also provide new mechanistic insight into the basis for habitat selection and how global change may impact birds at their nesting sites.

## Supplementary Material

Supplementary information

## Figures and Tables

**Table 1:** The outputs of logistic regression models predicting colony presence for four different initial wind directions. Three summary statistics were modelled for each section and wind property: ‘skewness’ (Skew), ‘median’ (Med) and ‘interquartile range’ (IQR). In addition, three colony classifications were tested, where colony presence was taken as 1) any positive count, 2) the 10 largest colonies and 3) the 11 densest colonies. Shelter from higher wind speeds is associated with low pressure (PMed) and reduced pressure range (PIQR). Exposure to higher wind speeds is highlighted by negative and decreased skewness in horizontal wind speed. Higher turbulence is indicated by increased TKE median and lowTKE skewness. TI terms follow the same pattern. Significance is indicated according to p-value: p < 0.001 (***), p < 0.01 (**), p < 0.005 (*) and model predictors are listed in order of descending effect size (Supporting information).

	SW			NW			SE			NE		
Colony definition	Term	p -value/significance	Est.	Term	p -value/significance	Est.	Term	p -value/significance	Est.	Term	p -value/significance	Est.
Any count	PMedian	<0.001***	−2.27	TISkew	< 0.01**	−1.94	TKEIQR	<0.05*	−1.36	TKESkew	<0.001***	−4.39
	TISkew	<0.05*	−1.54	TKESkew	<0.01**	−2.27	MeanSlope	<0.01**	+1.88	MeanSlope	<0.001***	+2.80
	MeanSlope	<0.05*	+1.49							HorizontalMedian	<0.01**	+2.12
10 Largest	PIQR	<0.05*	−6.23	TKESkew	< 0.01**	−5.96	MeanSlope	<0.001***	+3.59	TKEIQR	<0.01**	+7.75
	HorizontallQR	<0.01**	+5.65	MeanSlope	< 0.01**	+2.56				HorizontalSkew	<0.05*	−7.26
	MeanSlope	<0.001***	+4.08							MeanSlope	<0.01**	+4.53
11 Densest	PMedian	<0.05*	−2.50	TKESkew	< 0.05*	−4.10	MeanSlope	<0.05*	+1.95	TKEMedian	<0.01**	+8.27
				MeanSlope	< 0.05*	+1.92				U_2 Median	<0.01**	−4.23
										HorizontalSkew	<0.05*	−3.34
McFadden pseudo *R^2^* – OA/TSS/sensitivity/specificity												
Any count	0.28–0.66/0.32/0.63/0.69			0.24–0.63/0.21/1.00/0.21			0.12–0.56/0.18/0.21/0.96			0.38–0.71/0.45/0.60/0.84		
10 Largest	0.59–0.90/0.73/0.80/0.93			0.58–0.86/0.81/1.00/0.81			0.32–0.86/0.60/0.70/0.90			0.74–0.97/0.90/0.90/1.00		
11 Densest	0.17–0.65/0.48/0.90/0.57			0.40–0.84/0.78/1.00/0.78			0.15–0.72/0.51/0.81/0.69			0.67–0.88/0.84/1.00/0.84		

## Data Availability

Data are available from the Dryad Digital Repository: <http://dx.doi.org/10.5061/dryad.h9w0vt4jk> (Lempidakis et al. 2021).
