# Supplementary material for: Airflow modelling predicts seabird breeding habitat across islands
Source: Ecography. Author manuscript; Available in PMC 2022 Jan 4. (PMC7612159; doi:10.1111/ecog.05733)
Supplement: Supplementary information [file EMS140358-supplement-Supplementary_information.pdf]

Supporting information

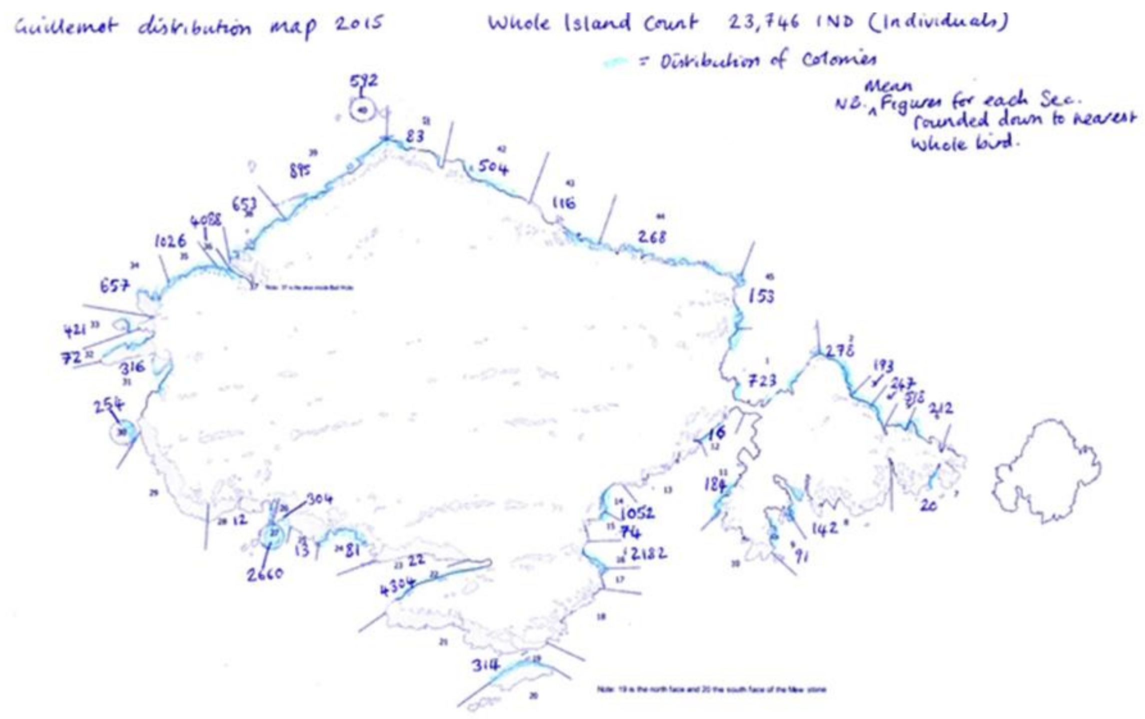

Figure S1. The guillemot survey map reproduced from the 2015 Guillemot survey data on Skomer. The 45 sections presented, were sub- divided into a total of 71 sections, with the blue shades indicating the horizontal areas that the birds are located.

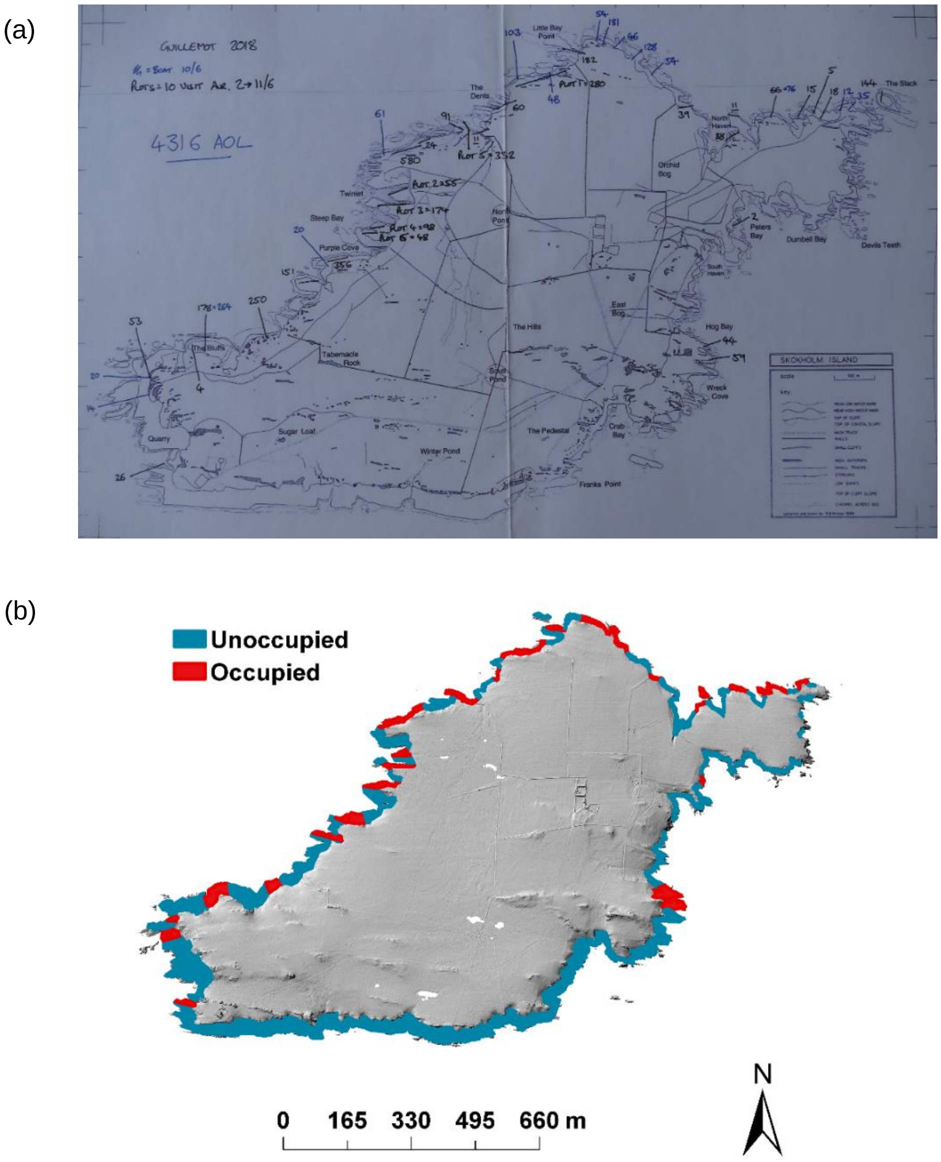

Figure S2. A) The guillemot survey map reproduced from the 2018 Guillemot survey data on Skokholm and B) the resulting digitised sections with blue indicating unoccupied and red occupied cliffs.

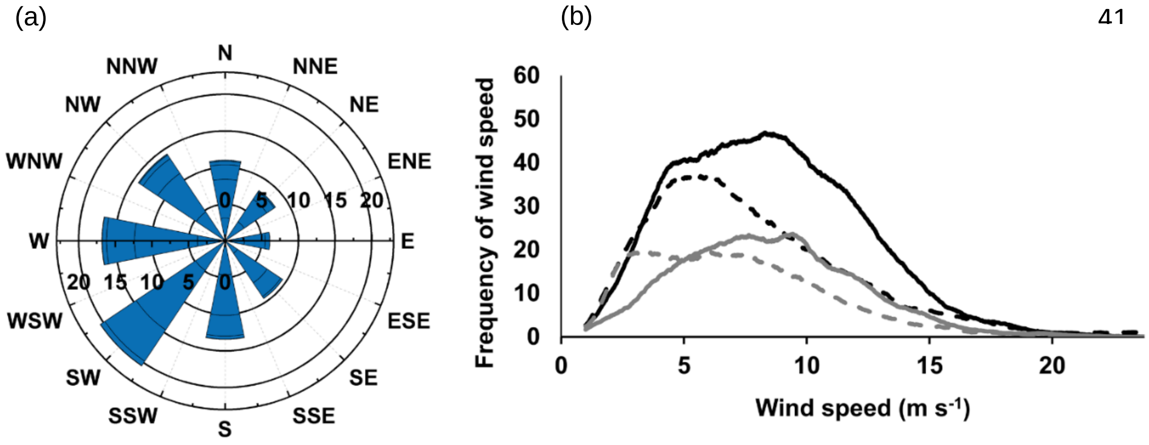

Figure S3. Wind speeds and directions during auk breeding seasons for 2007 to 2017. A) Overall frequency of wind directions on Skomer (2007-2017) , B) The frequency of average wind speed, given per 100 observations across the four different wind directions that were modelled: SW with solid black, NW with dashed black, SE with solid grey and NE with dashed grey, lines.

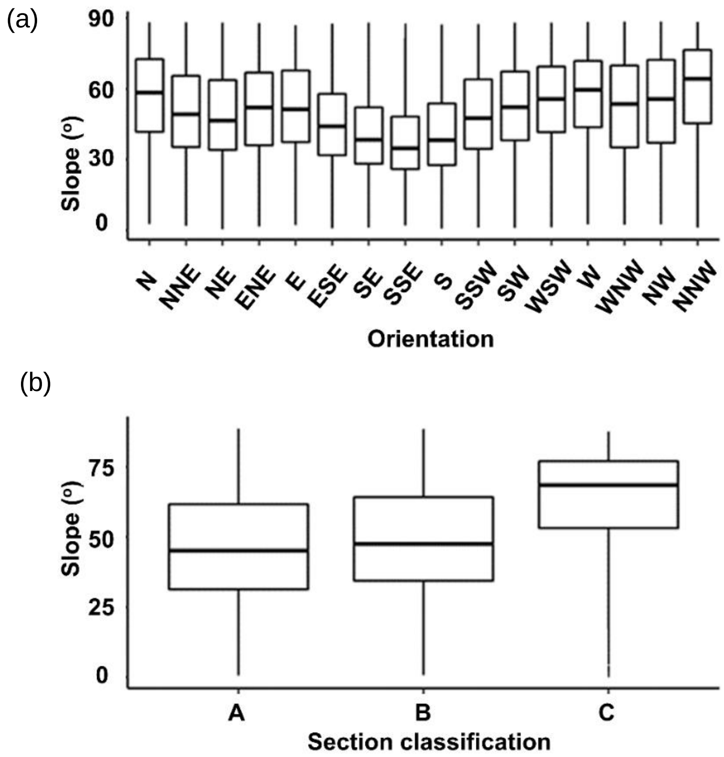

Figure S4. Slope angles selected by breeding birds in relation to those available on Skomer. A) Mean slope angle (^{o}) according to cliff orientation (number of points per orientation bin; 2911- 8718). B) Slope angle for (A) unoccupied sections (n=33, median= ~45.1^{o}), (B) occupied but not classified as either largest or densest sections (n=31, median= ~47.5^{o}) and (C) sections classified as both largest and densest (n=7, median= 68.5^{o}).

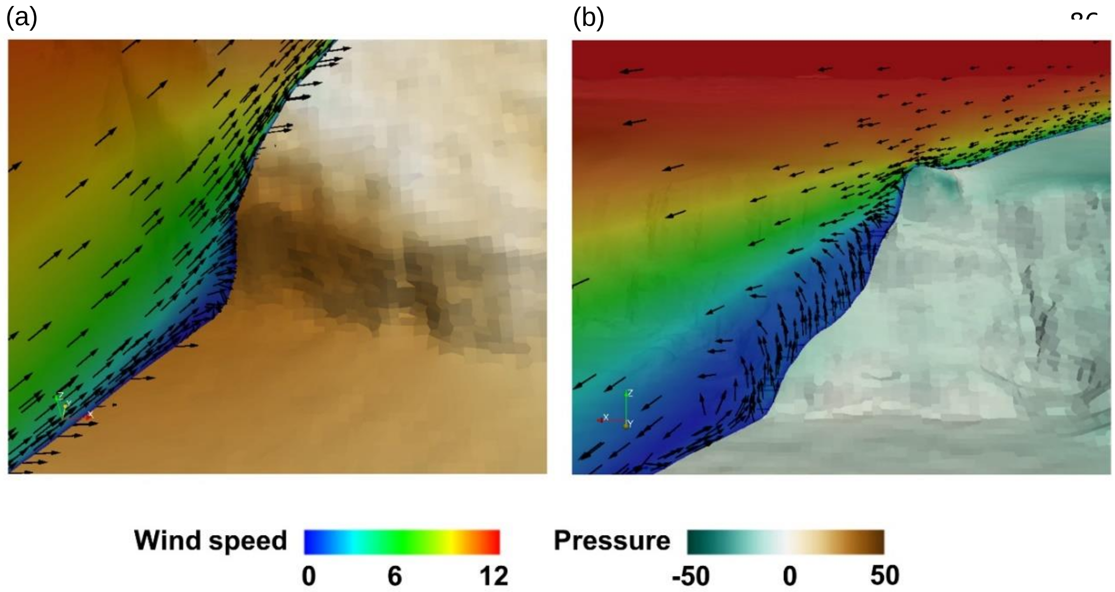

Figure S5. Wind speed and pressure over A) a windward cliff and B) a leeward cliff on Skomer, in a SW wind. In each case the cliff face is the section to the right, which is represented in 3 dimensions and coloured according to the gauge pressure (that is, relative to the initial condition set in OpenFoam) distribution 2 m normal to the surface. The associated 2-dimensional vertical profile of mean wind speed is indicated in colour to the left of the cliff. Arrows indicate the mean flow vectors. Together, these illustrate how the flow onto a windward cliff result in high pressure, and low pressure develops in sheltered leeward sites.

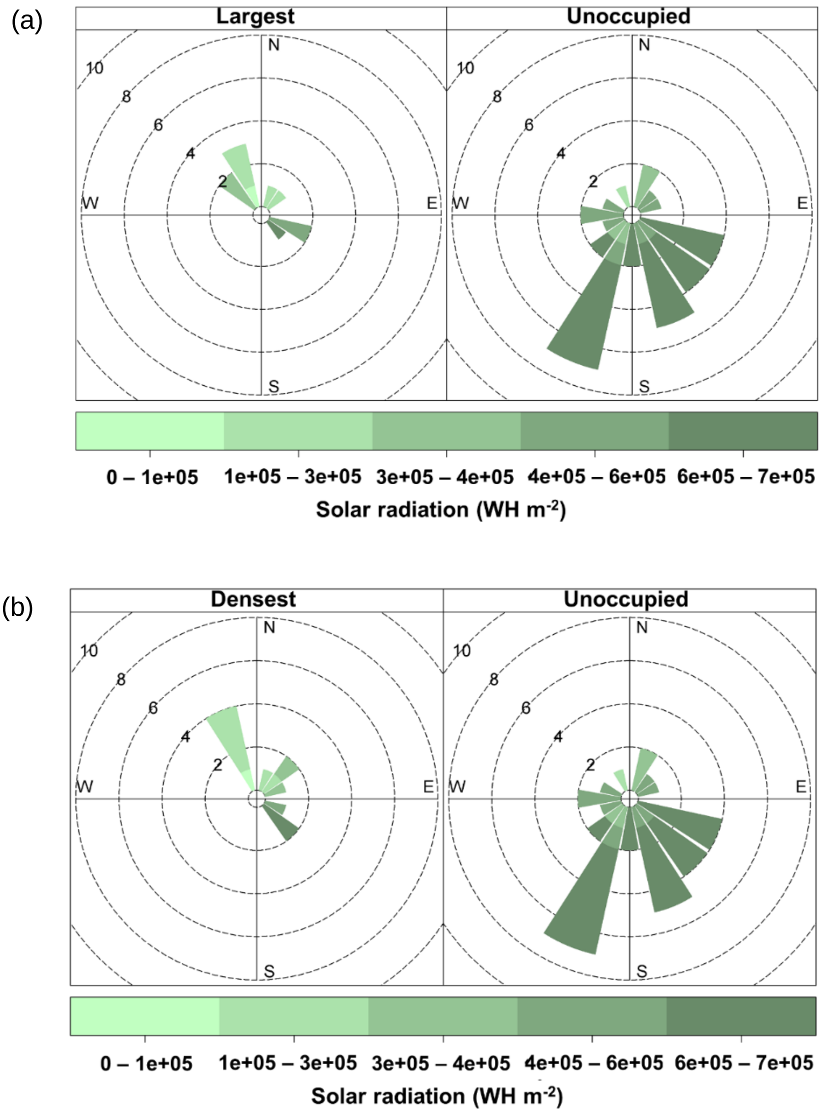

Figure S6. Total mean solar radiation during the breeding season in 2015 (Watt hours sq. m) on Skomer, according to whether cliff sections were home to the a) largest colonies, b) densest colonies or unoccupied. Grid circles represent number of cliffs/ colonies, with radiation intensity indicated by the colour scale.

Table S1. Wind data for the months of March to August 2007-2017, representing the guillemot breeding season. Data are from the weather station at Wooltack point on the mainland (51° 42.581'N 5° 3.786'W), ~1 km from Skomer Island.

**Direction**	**Count**	**Wind speeds <15 m s^{-1}**	**Wind speeds ≥15 m s^{-1}**	**% near gale winds within each group**	**% near gale winds from total obs**	**% near gale winds from total storms**	**Group direction % from total wind obs**
**SW**	46096	44356	1740	3.77	0.77	20.36	20.52
**W**	37800	36365	1435	3.8	0.64	16.79	16.83
**NW**	32052	30308	1744	5.44	0.78	20.4	14.27
**S**	30223	29210	1013	3.35	0.45	11.85	13.46
**N**	24877	23490	1387	5.58	0.62	16.23	11.08
**SE**	21253	20538	715	3.36	0.32	8.37	9.46
**NE**	18573	18105	468	2.52	0.21	5.48	8.27
**E**	13746	13701	45	0.33	0.02	0.53	6.12

Table S2. Effect sizes for the Skomer habitat selection model, expressed as odds ratios. Odds ratios > 1 were estimated for predictors with a positive coefficient, odds ratios <1 were estimated for predictors with a negative coefficient, and odds ratios close to 1 were the result of predictors with a relatively small effect on the probability of a section being classified as a colony. Model predictors are listed in order of descending effect size.

	**SW**		**NW**		**SE**		**NE**	
**Colony definition**	**Term**	**Odds ratio**	**Term**	**Odds ratio**	**Term**	**Odds ratio**	**Term**	**Odds ratio**
**Any count**	PMedian<br>TISkew<br>MeanSlope	1.0e-01<br>2.1e-01<br>4.5e+00	TISkew<br>TKESkew	1.4e-01<br>1.0e-01	TKEIQR<br>MeanSlope	2.5e-01<br>6.6e+00	TKESkew<br>MeanSlope<br>HorizontalMedian	1.2e-02<br>1.7e+01<br>8.3e+00
**Largest**	PIQR<br>HorizontalIQR<br>MeanSlope	2.0e-03<br>2.9e+02<br>5.9e+01	TKESkew<br>MeanSlope	2.6e-03<br>1.3e+01	MeanSlope	3.6e+01	TKEIQR<br>HorizontalSkew<br>MeanSlope	2.3e+03<br>7.0e-04<br>9.3e+01
**Densest**	PMedian	8.1e-02	TKESkew<br>MeanSlope	1.6e-02<br>6.9e+00	MeanSlope	7.1e+00	TKEMedian<br>U_2Median<br>HorizontalSkew	3.9e+03<br>1.4e-02<br>3.5e-02

Sensitivity analysis

Table S3. The sensitivity analysis of the top simplest model terms for different thresholds of number of birds and number of largest colonies, with the prevailing SW wind. Significance is indicated with p-values: $p < 0.001$ (***), $p < 0.01$ (**), $p < 0.005$ (*).

Largest colonies / Number of birds threshold	Top simplest model - terms	McFadden pseudo R^2 – OA/ TSS/ Sensitivity/ Specificity
10/592	PIQR (*), HorizontalIQR (**), MeanSlope (***)	0.59 – 0.90/ 0.73/ 0.80/ 0.93
11/518	PIQR (*), HorizontalIQR (**), MeanSlope (***)	0.57 – 0.81/ 0.75/ 1.00/ 0.75
12/504	PIQR (*), HorizontalIQR (*), MeanSlope (***)	0.54 – 0.80/ 0.72/ 1.00/ 0.72
13/421	PIQR (n.s.), MeanSlope (*), PMedian (**), U_2IQR (**)	0.55 – 0.84/ 0.74/ 0.92/ 0.81
14/316	PIQR (*), MeanSlope (*), PMedian (**), U_2IQR (**)	0.52 – 0.82/ 0.67/ 0.85/ 0.81
15/314	PIQR (*), HorizontalIQR (n.s.), MeanSlope (**), PMedian (**), U_2IQR (**), U_2Skew (*)	0.64 – 0.93/ 0.87/ 0.93/ 0.93

Table S4. The sensitivity analysis of the top simplest model terms for different thresholds of number of birds and number of largest colonies, with NW wind. Significance is indicated with p-values: p< 0.001 (***), p< 0.01 (**), p< 0.005 (*).

**Largest colonies / Number of birds threshold**	**Top simplest model - terms**	**McFadden pseudo R^2 – OA/ TSS/ Sensitivity/ Specificity**
10/592	TKESkew (**), MeanSlope (**)	0.58 – 0.86/ 0.81/ 1.00/ 0.81
11/518	TKESkew (**), MeanSlope (**)	0.59 – 0.86/ 0.81/ 1.00/ 0.81
12/504	TKESkew (**), MeanSlope (**)	0.55 – 0.84/ 0.78/ 1.00/ 0.78
13/421	TKESkew (***), MeanSlope (*)	0.55 – 0.82/ 0.75/ 1.00/ 0.75
14/316	TKESkew (***), MeanSlope (**)	0.50 – 0.80/ 0.68/ 0.92/ 0.75
15/314	TKESkew (***), MeanSlope (**)	0.50 – 0.77/ 0.66/ 1.00/ 0.66

Table S5. The sensitivity analysis of the top simplest model terms for different thresholds of number of birds and number of largest colonies, with NE wind. Significance is indicated according to p-value: $p< 0.001$ (***), $p< 0.01$ (**), $p< 0.005$ (*).

Largest colonies / Number of birds threshold	Top simplest model - terms	McFadden pseudo R^2 – OA/ TSS/ Sensitivity/ Specificity
10/592	TKEIQR (**), HorizontalSkew (*), MeanSlope (**)	0.74 – 0.97/ 0.90/ 0.90/ 1.00
11/518	TKEIQR (**), HorizontalSkew (*), MeanSlope (**)	0.76 – 0.97/ 0.90/ 0.90/ 1.00
12/504	TKEIQR (**), HorizontalSkew (*), MeanSlope (**)	0.74 – 0.95/ 0.88/ 0.91/ 0.96
13/421	HorizontalSkew (*), TKEMedian (***), U_2Median (***)	0.67 – 0.80/ 0.72/ 1.00/ 0.72
14/316	HorizontalMedian (**), TKESkew (**), MeanSlope (***)	0.57 – 0.80/ 0.68/ 0.92/ 0.75
15/314	HorizontalMedian (**), TKESkew (**), MeanSlope (***)	0.59 – 0.89/ 0.70/ 0.73/ 0.96

Model of cliff orientation and slope angle

For the implementation of the mean cliff orientation and slope angle models, the same model selection was performed as for the airflow models, as described in the main text.

Table S6. Logistic regression models of leeward/ windward cliff orientation with respect to the prevailing SW wind on Skomer.

**Colony definition**	**Orientation model:**<br><br>**McFadden pseudo R^2 – OA/ TSS/ Sensitivity/ Specificity**
Any count	Aspect + MeanSlope<br><br>0.17 – 0.63/ 0.30/ 0.42/ 0.87
10 largest	Aspect + MeanSlope<br><br>0.55 – 0.88/ 0.71/ 0.80/ 0.90
11 densest	Aspect<br><br>0.20 – 0.68/ 0.51/ 0.90/ 0.60
